# Cognitive performance and dental care utilization and dental hygiene practice among older adults

**DOI:** 10.1093/geroni/igaf122.3335

**Published:** 2025-12-31

**Authors:** Huabin Luo, Xiang Qi, Ruotong Mona Liu, Bei Wu

**Affiliations:** East Carolina University, Greenville, North Carolina, United States; New York University, New York, New York, United States; New York University, New York, New York, United States; NYU Shanghai, Shanghai, China (People’s Republic)

## Abstract

Dental care is crucial for oral health and there is growing evidence that poor oral health contributes to cognitive decline. However, little research has assessed the relationship between different domains of cognitive function and dental utilization and dental self-care. Using National Health and Nutrition Examination Survey (NHANES) 2011-2014, we investigated the associations of cognitive performance with dental utilization and self-care. The sample included 2,495 adults aged 60 years or older and having at least one tooth. Cognitive performance was assessed by the Consortium to Establish a Registry for Alzheimer’s Disease: immediate word recall (IWR), delayed recall (DR), Animal Fluency Test (AFT), and Digit Symbol Substitution Test (DSST). We also calculated a low cognitive performance score based on education levels, defined as 1 SD below the mean on a composite z-score of these four tests. The three outcome variables were dental visits, preventive dental visits, and dental flossing. Multivariate regression model results showed that higher levels of cognitive performance in DR and DSST were associated with more frequent dental visits (all P<.05). In addition, low levels of IWR, AFT, and DSST were significantly associated with less dental flossing (all P<.05). Overall low cognitive performance was associated with less dental flossing (AOR=0.48, P=.02). These results suggest that older adults with low cognitive performance are less likely to have regular dental visits or conduct proper dental hygiene practice. Oral health awareness and oral health literacy should be further promoted among older adults and family members.

